# Diffuse Neurofibroma of the Scalp Presenting as Circumscribed Alopecic Patch

**DOI:** 10.4103/0974-7753.66919

**Published:** 2010

**Authors:** BC Sharath Kumar, MG Gopal, Ankur Talwar, M Ramesh

**Affiliations:** Department of Dermatology, Kempegowda Institute of Medical Sciences Hospital, Bangalore, India

**Keywords:** Alopecia Areata, diffuse neurofibroma, scalp

## Abstract

Neurofibroma is a benign tumor of the peripheral nerve sheath characterized by proliferation of Schwann cells, perineural cells and endoneurial fibroblasts. Different types of neurofibromas can be identified, including localized, plexiform, and diffuse types. Diffuse neurofibroma is an uncommon form of neurofibroma that occurs primarily in children and young adults. The head and neck regions are the most common sites of involvement. Diffuse neurofibroma is an ill-defined infiltrative lesion and tends to involve the skin and subcutaneous tissues. It produces localized thickening and induration of the skin. We present a case of a 12-year-old boy who had a diffuse neurofibroma on the scalp since the age of 2 years.

## INTRODUCTION

Diffuse neurofibroma is a newly recognized distinct clinical entity. Diffuse neurofibroma is an uncommon, but distinct, variety of neurofibroma usually affecting the trunk, head, and neck regions of adolescents and young adults. It is not clear exactly how often diffuse neurofibromas are associated with neurofibromatosis, although it has been suggested that about 10% of patients with diffuse neurofibromas have neurofibromatosis 1 (NF1, von Recklinghausen’s disease).[1,2]

There are very few previously reported cases of diffuse neurofibroma of the scalp in the Indian literature. We report a case of a diffuse neurofibroma on the scalp of a young boy, which was confirmed by radiologic imaging and histopathologic examination. The case is reported for its rarity and its unique presentation.

## CASE REPORT

**Figure 1:**
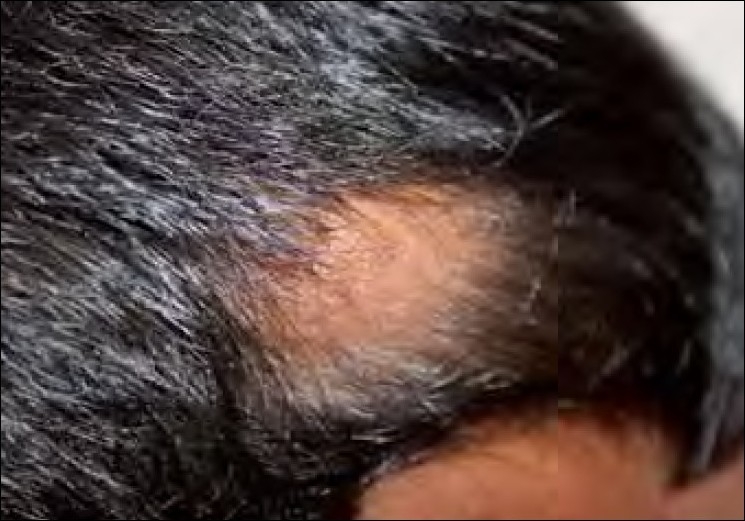
A 12-year-old boy with circumscribed alopecic patch on the scalp

**Figure 2:**
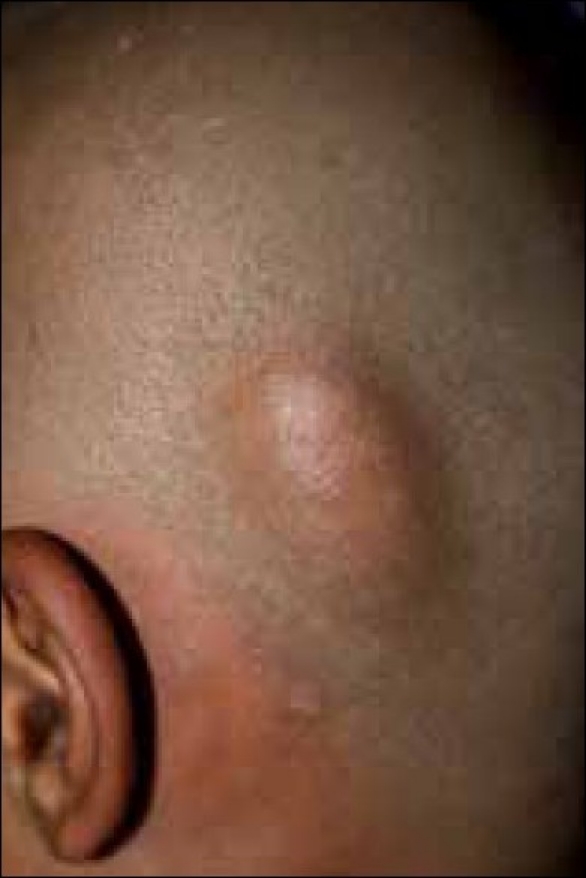
Close-up view of the lesion on shaving the scalp. There were no bony depressions; however, the mass had a bag of worms feel on palpation

A 12-year-old boy was seen at our outpatient clinic because of a bulging mass with loss of overlying hair on the posterior aspect of his scalp. The mass was first observed at the age of 2 years, and it had slowly increased in size since then. Physical examination revealed a soft 4 × 4 cm skin colored mass with mild tenderness on the occipital scalp [Figure 1]. There was loss of hair on the skin overlying the mass. The mass had a ‘bag of worms’ feel on palpation. Neither were there any changes in the bone around the margins of the swollen mass [Figure 2] nor was there any bruit over the mass. The rest of the cutaneous examination was unremarkable and failed to reveal any *cafe-au-lait* spots or axillary/inguinal freckling. An ophthalmologic opinion was also sought for which did not reveal any abnormality.

At the time, the tentative differential diagnoses included Alopecia Areata, hemangioma, lipoma, and eccrine spiradenoma.

Plain skiagram of the skull showed no bony erosion. An ultrasound was performed, which showed a hypoechoic mass suggestive of hemangioma. A computed tomography (CT) scan performed subsequently failed to reveal any intracranial extension. Rest of the laboratory data was normal.

**Figure 3:**
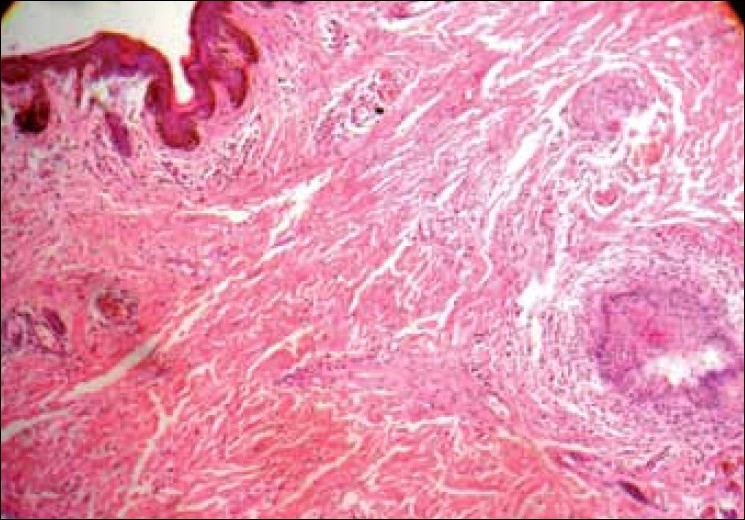
Histopathology picture (10×) showing proliferation of spindle cells in the dermis with an unremarkable epidermis

**Figure 4:**
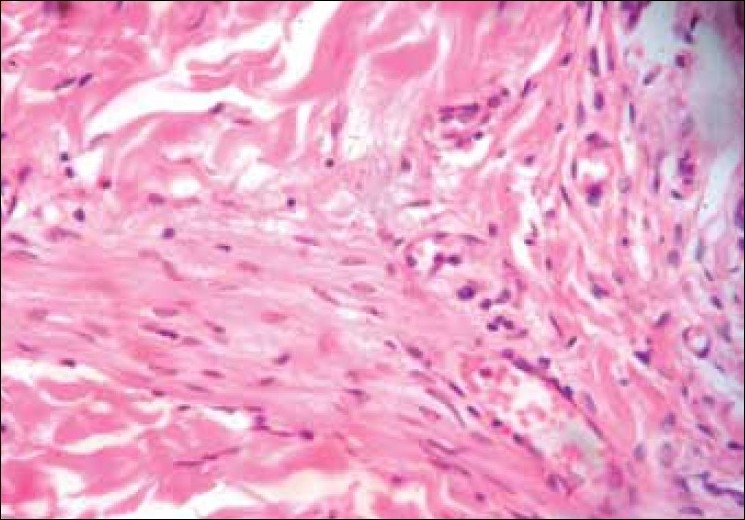
Histopathology picture (40×) showing the multiple spindleshaped cells with elongated ovoid to curved nuclei, surrounded by ecstatic blood vessels a matrix with wire like collagenous fibers

The patient subsequently underwent surgery for wide excision of the mass. Histologic examination of the excised specimen [Figure 3] showed a typical presentation of diffuse neurofibroma involving the entire dermis and infiltrating the subcutaneous fat. The lesion was characterized by a proliferation of spindle cells that contained elongated ovoid to curved nuclei, and these cells were surrounded by a matrix with wire like collagenous fibers. Few ecstatic blood vessels were also found [Figure 4].

The tumor was completely excised. No recurrence was observed during the first year of follow-up. The patient’s physical and mental development showed no abnormalities, and a diagnosis of Von Recklinghausen’s neurofibromatosis could not be established.

## DISCUSSION

Neurofibromas are benign tumors of the peripheral nerves that have a neuroectodermal origin. On the basis of the histology, three types of neurofibroma can be distinguished: the localized, plexiform, and diffuse types.[3] The localized neurofibroma presenting as a fusiform or ovoid mass involves the nerve, which may be seen entering and exiting the tumor mass. The plexiform neurofibroma diffusely involves a long segment of nerve and its branches and shows a serpentine like appearance. The diffuse neurofibroma is characterized by infiltrative growth in the subcutaneous fat and entrapment of the fat and other normal structures.[4]

The essential cells in neurofibromas are of Schwann-cell origin.[5] Diffuse neurofibroma is an uncommon, but distinctive, form of neurofibroma. It is variably sized, though often large. It is characterized by marked dermal and subcutaneous thickening that most often appears in the trunk or head and neck regions of adolescents or young adults.[6] This lesion has also been termed ‘paraneurofibroma’.[5] Intracranial extension of the extracranial variety of this tumor has rarely been reported.[7] At least 10% of these tumors is associated with NF-1.

On histopathologic examination, the tumor is composed of elongated, spindle-shaped cells with round or fusiform nuclei and eosinophilic cytoplasm within a loose matrix of fine fibrillary collagen. Meissner bodies are characteristic, but are not always present. Neurofibromatous tissues show immunoreactivity with S-100 protein, a sensitive, but non-specific, marker of benign nerve sheath tumors.[8]

The treatment of large neurofibromas consists of partial or complete surgical excision. Even after complete excision, clinical recurrence may develop because of the infiltrative growth pattern of the tumor. Because of possible recurrence, yearly follow-up is recommended.

Because of the lack of distinctive clinical characteristics, especially in isolated lesions, it is difficult to diagnose the lesion preoperatively, and such lesions, remain largely, a histopathologic diagnosis. Proper history-taking, physical examination, opthalmologic and radiodiagnostic investigations are warranted in such cases, with special attention to detection of *cafe-au-lait* spots, Lisch nodules, and bilateral acoustic to exclude neurofibromatoses.

Thus, diffuse neurofibroma should always be kept in mind in the differential diagnosis of any indurated mass on the scalp. The present case has been presented for its rarity and its unique presentation.
